# Structure-specific nucleases in genome dynamics and strategies for targeting cancers

**DOI:** 10.1093/jmcb/mjae019

**Published:** 2024-05-07

**Authors:** Haitao Sun, Megan Luo, Mian Zhou, Li Zheng, Hongzhi Li, R Steven Esworthy, Binghui Shen

**Affiliations:** Medicinal Plant Resources and Protection Research Center, Institute of Medicinal Plant Development, Chinese Academy of Medical Sciences, Peking Union Medical College, Beijing 100193, China; Department of Cancer Genetics and Epigenetics, Beckman Research Institute of City of Hope, Duarte, CA 91010, USA; Department of Cancer Genetics and Epigenetics, Beckman Research Institute of City of Hope, Duarte, CA 91010, USA; Department of Cancer Genetics and Epigenetics, Beckman Research Institute of City of Hope, Duarte, CA 91010, USA; Department of Cancer Genetics and Epigenetics, Beckman Research Institute of City of Hope, Duarte, CA 91010, USA; Department of Cancer Genetics and Epigenetics, Beckman Research Institute of City of Hope, Duarte, CA 91010, USA; Department of Cancer Genetics and Epigenetics, Beckman Research Institute of City of Hope, Duarte, CA 91010, USA; Department of Cancer Genetics and Epigenetics, Beckman Research Institute of City of Hope, Duarte, CA 91010, USA

**Keywords:** structure-specific nucleases, DNA replication, nuclease inhibitors, synthetic lethality

## Abstract

Nucleases are a super family of enzymes that hydrolyze phosphodiester bonds present in genomes. They widely vary in substrates, causing differentiation in cleavage patterns and having a diversified role in maintaining genetic material. Through cellular evolution of prokaryotic to eukaryotic, nucleases become structure-specific in recognizing its own or foreign genomic DNA/RNA configurations as its substrates, including flaps, bubbles, and Holliday junctions. These special structural configurations are commonly found as intermediates in processes like DNA replication, repair, and recombination. The structure-specific nature and diversified functions make them essential to maintaining genome integrity and evolution in normal and cancer cells. In this article, we review their roles in various pathways, including Okazaki fragment maturation during DNA replication, end resection in homology-directed recombination repair of DNA double-strand breaks, DNA excision repair and apoptosis DNA fragmentation in response to exogenous DNA damage, and HIV life cycle. As the nucleases serve as key points for the DNA dynamics, cellular apoptosis, and cancer cell survival pathways, we discuss the efforts in the field in developing the therapeutic regimens, taking advantage of recently available knowledge of their diversified structures and functions.

## Introduction

**Figure 1:**
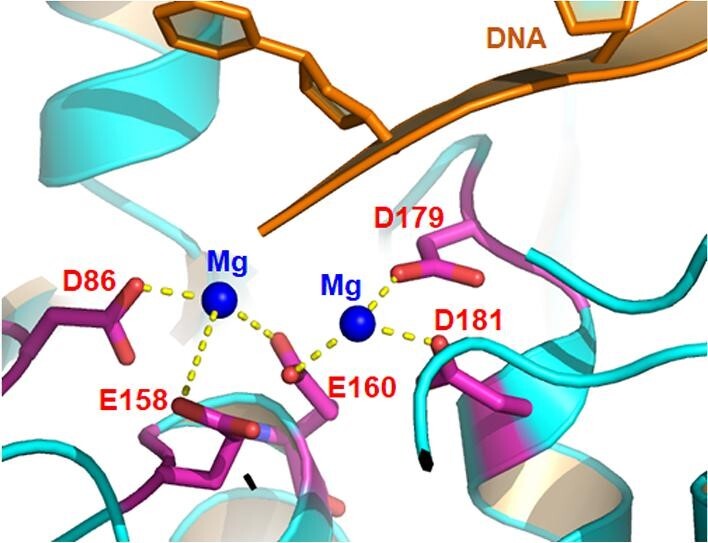
Diagram to show the relative positions of the nuclease catalytic centres of FEN1 and the DNA substrate. The FEN1’s active centre is composed of a pair of magnesium atoms displayed as blue balls, which is coordinated by the metal-binding acidic amino acid residues in purple (Ds and Es). The DNA molecule is coloured in brown. This image was captured from PyMOL and labelled with photoshop.

Nucleases are responsible for catalyzing the cleavage of phosphodiester bonds between nucleotides. The cleavage patterns have two action orientations, 3′ and 5′, and vary widely in substrates. Nucleases distinguishing into two main types; ‘exonucleases’ cleave a single nucleotide off the end of a substrate, while ‘endonucleases’ create a nick in the middle of a DNA or RNA strand. Throughout evolution, nucleases have evolved to be sequence specific and recognize substrates through hydrogen bond interactions with nitrogenous bases (Bower et al., 2018; Alonso-Lerma et al., 2023). Eukaryotic structure-specific nucleases help to maintain genome stability as they play important roles in DNA replication, repair, recombination, and apoptotic DNA fragmentation (Table 1). In contrast to the sequence-specific recognition model of restriction enzymes and the RNA-guided or homing sequence recognition for the CRISPR/Cas system of bacteria, almost all eukaryotic nucleases have a structure- or conformation-specific substrate recognition model. While the cleavage mechanism of structure-specific nucleases has been elucidated and is well understood, more research is needed to understand how they differentiate alternative structures. The functional collective of nucleases is highly diverse with little sequence or structure similarity. However, most nucleases utilize the same two-divalent metal ion catalysis mechanism. The divalent cation, often Mg^++^, will act as a Lewis base and attract electron density to facilitate a more favourable nucleophilic attack. Seven to eight conserved acidic residues, such as aspartate and glutamate, anchor the cations and form a catalytic centre (Figure 1). Secondary, the cation also serves to neutralize the concentration of negative charges of the phosphodiester bond and thereby providing stability (Yang, 2011). Nucleases like DNA replication helicase/nuclease 2 (DNA2), EXO5, and endonuclease III-like protein 1 (NTHL1) have Fe–S centres that act as part of a selective tunnel, allowing only a single strand of DNA to enter the site for actual metal cation-catalyzed hydrolysis (Zhou et al., 2015).

**Table 1:** Structure-specific nucleases that edit the genomes and nucleases that process RNA.

		**Nucleases in model organisms**					
**Biological functions**	**Biochemical activities**	***Homo sapiens***	***Mus musculus***	***Drosophila melanogaster***	***C. elegans***	***S. cerevisiae***	***E. coli***
DNA replication •Proofreading •RNA primer removal •Generation of telomere T-loop •Resolution of D-loop	•3′ exonuclease •5′ exonuclease •3′ flap endonuclease •5′ flap endonuclease	DCLRE1A	Dclre1a	Snm1	SNM1A	Pso2	–
		DCLRE1B	Dclre1b	–	MRT-1	Pso2	–
		DNA2	Dna2	CG2990	DNA-2	Dna2	–
		FEN1	Fen1	Fen1	CRN-1	Rad27	Pol I
		Pol ε	Pol ε	Pol ε	Pol ε	Pol ε	Pol III
		Pol δ	Pol δ	Pol δ	Pol δ	Pol δ	Pol III
		Pol γ	Pol g	PolG1	POLG-1	Mip1	Pol I
		ZRANB3	Zranb3	–	–	–	–
		EXO1	Exo1	tos	EXO-1	Din7	–
		MIF	Mif	–	MIF-1	–	–
DNA repair •Incision •Excision	•Nick endonuclease •3′ exonuclease •5′ exonuclease •3′ flap endonuclease •5′ flap endonuclease	APE1	Apex1	Rrp1	EXO-3	Apn2	Exo III
		APE2	Apex2		EXO-3	Apn2	Exo III
		EXO1	Exo1	tos	EXO-1	Exo1	–
		FAN1	Fan1		FAN-1	–	–
		FEN1	Fen1	Fen1	CRN-1	Rad27	TatD
		GEN1	Gen1	Gen	GEN-1	Yen1	–
		NTHL1	Nthl1	CG9272	NTH-1	Ntg1/Ntg2	Endo III
		MLH1	Mlh1	Mlh1	MLH-1	Mlh-1	MutL
		MRE11	Mre11a	mre11	MRE-11	Mre11	SbcCD
		Pol β	Pol β	–	Pol β	Pol β	Pol I
		Pol δ	Pol δ	Pol δ	Pol δ	Pol δ	Pol III
		TREX1	Trex1	CG3165	W02F12.4	–	RNase T
		TREX2	Trex2	CG3165	W02F12.4	–	RNase T
		XPF/ERCC1	Ercc4/Ercc1	mei-9/Ercc1	XPF-1/ERCC-1	Rad1/Rad10	UvrC
		XPG	Ercc5	mus201	XPG-1	Rad2	–
		ASTE1	Aste1	ast	–	–	–
		–	–	–	APN-1	Apn1	Endo IV
		RAD1	Rad1	Rad1	MRT-2	Rad17	–
		EME1	Eme1	mms4	–	Mms4	–
DNA recombination •End resection •Resolution of Holliday junction •Resolution of stalled replication fork •V(D)J recombination	•3′ exonuclease •5′ exonuclease •3′ flap endonuclease •5′ flap endonuclease •Holliday junction resolvase •Gap-dependent endonuclease	DCLRE1C	Dclre1c	–	–	Pso2	Exo I
		CtIP	Rbbp8	CtIP	COM-1	Sae2	–
		DNA2	Dna2	CG2990	DNA-2	Dna2	–
		XPF/ERCC1	Ercc4/Ercc1	mei-9/Ercc1	XPF-1/ERCC-1	Rad1/Rad10	UvrC
		EXO1	Exo1	tos	EXO-1	Exo1	–
		FEN1	Fen1	Fen1	CRN-1	Rad27	Pol I
		GEN1	Gen1	Gen	GEN-1	Yen1	–
		Mre11	Mre11	mre11	MRE-11	Mre11	Mre11
		MUS81/EME1	Mus81/Eme1	mus81/mms4	MUS-81/EME-1	Mus81/Mms4	–
		RAG1/RAG2	Rag1/Rag2	Transib	–	–	–
		SLX1A	Slx1b	slx1	SLX-1	Slx1	–
		SLX4	Slx4	mus312	SLX-4	Slx4	–
		SPO11	Spo11	mei-W68	SPO-11	Spo11	–
Apoptosis •DNA fragmentation	•Nick endonuclease •DNA exonuclease •Gap-dependent endonuclease	DFF40/CAD	DFF40/CAD	Drep-4/CAD	–	–	–
		DNase I	DNase I	DNase I	DNase I	–	–
		DNase IIa	Dnase2a	DNase II	NUC-1	–	–
		DNase llb	–	–	CRN-6/CRN-7	–	RecA
		ENDOG	Endog	EndoG	CPS-6	Nuc1	NucA
		FEN1	Fen1	Fen1	CRN-1	Rad27	DNase I
		TATDN1	Tatdn1	CG3358	CRN-2/TAT-D	Tat-D	–
		ERN2	Ern2	Ire1	IRE-1	Ire1	–
RNA metabolism •mRNA degradation •rRNA processing •siRNA processing •RNA:DNA hybrid degradation	•3′ exonuclease •5′ exonuclease • endonuclease	DIS3	DIS3	Dis3	dis-3	Dis3	–
		ERI1	Eri1	Snp	R02d3.8	–	–
		EXOSC10	Exosc10	Rrp6	crn-3	Rrp6	–
		RNASEH1	Rnaseh1	Rnh1	rnh-1.0	Rnh1	rnhA
		RNASEH2	Rnaseh2	Dmel/rnh1	–	Rnh2Ap	rnhB
		TREX1	Trex1	CG3165	W02F12.4	–	RNase T
		XRN1	Xrn1	Pcm	xrn-1	Xrn1	–
		XRN2	Xrn2	Rat1	xrn-2	Rat1	–
HIV life cycle							
•Processing of central DNA flap	•5′ flap endonuclease	FEN1	Fen1	Fen1	CRN-1	Rad27	Pol I

ASTE1, asteroid homologue 1; DCLRE1A/B/C, DNA cross-link repair 1A/B/C protein; DIS3, DIS3 homologue; ERN2, serine/threonine-protein kinase/endoribonuclease IRE2; MIF, macrophage migration inhibitory factor; MLH1, DNA mismatch repair protein Mlh1; MUS201, mutagen-sensitive 201; MUS312, mutagen-sensitive 312; RAD1, RAD1 checkpoint DNA exonuclease; RAG1/2, V(D)J recombination-activating protein 1/2; RTEL1, regulator of telomere elongation helicase 1; SPO11, SPO11 initiator of meiotic double-stranded breaks; ZRANB3, DNA annealing helicase and endonuclease.

The structure-specificity nature of the nucleases is exemplified by the founding member, flap endonuclease 1 (FEN1), of the family. Structural and functional analyses revealed the FEN1’s structure-specific, sequence-independent recognition for nicked double-stranded DNA (dsDNA) bent 100° with unpaired 3′ and 5′ flaps. Above the active site, a helical cap over a gateway formed by two helices enforced single-stranded DNA (ssDNA) threading and specificity for free 5′ ends. It is now known that dsDNA binding and bending, the ssDNA gateway, and double-base unpairing flanking the scissile phosphate control precise flap incision by two-metal-ion active site. Superfamily conserved motifs bind and open dsDNA; direct the target region into the helical gateway, permitting only non-base-paired oligonucleotides active site access; and support a unified understanding of the substrate recognition and specificity (Tsutakawa et al., 2011). The structural basis of the FEN1–PCNA interaction was also revealed by the crystal structure determination of the complex between human FEN1 and PCNA. The main interface involves the C-terminal tail of FEN1, which forms two β-strands connected by a short helix, the βA–αA–βB motif, participating in β–β and hydrophobic interactions with PCNA. The interactions at the interfaces maintain the enzyme in an inactive ‘locked-down’ orientation and might be utilized in rapid DNA-tracking by preserving the central hole of PCNA for sliding along the DNA. A hinge region present between the core domain and the C-terminal tail of FEN1 would play a role in switching the FEN1 orientation from an inactive to an active orientation that facilitates the flap DNA recognition and binding (Sakurai et al., 2005). Regardless of significant efforts spent to identify the structural elements to recognize the substrate configuration, it is still mysterious why some nucleases only recognize 5′ flap, while the others only recognize 3′ flap. Comparison by overlaying multiple nuclease structures in one category and many in the other category may give us insightful information to explain such a disparity.

The diversified functions make the nucleases essential to maintaining genome integrity and evolution in normal and cancer cells. In this article, we review their roles in various pathways from Okazaki fragment maturation (OFM) during DNA replication, end resection in homology-directed recombination repair (HDR) of DNA double-strand breaks (DSBs), to nucleotide excision in response to exogenous DNA damage. As the nucleases serve as key points for DNA dynamics, cellular apoptosis and cancer cell survival pathways, we discuss the efforts in the field in developing the therapeutic regimens, taking advantage of their diversified structures and functions.

## Structure-specific nucleases at the interface of OFM and DNA DSB recombination repair

**Figure 2:**
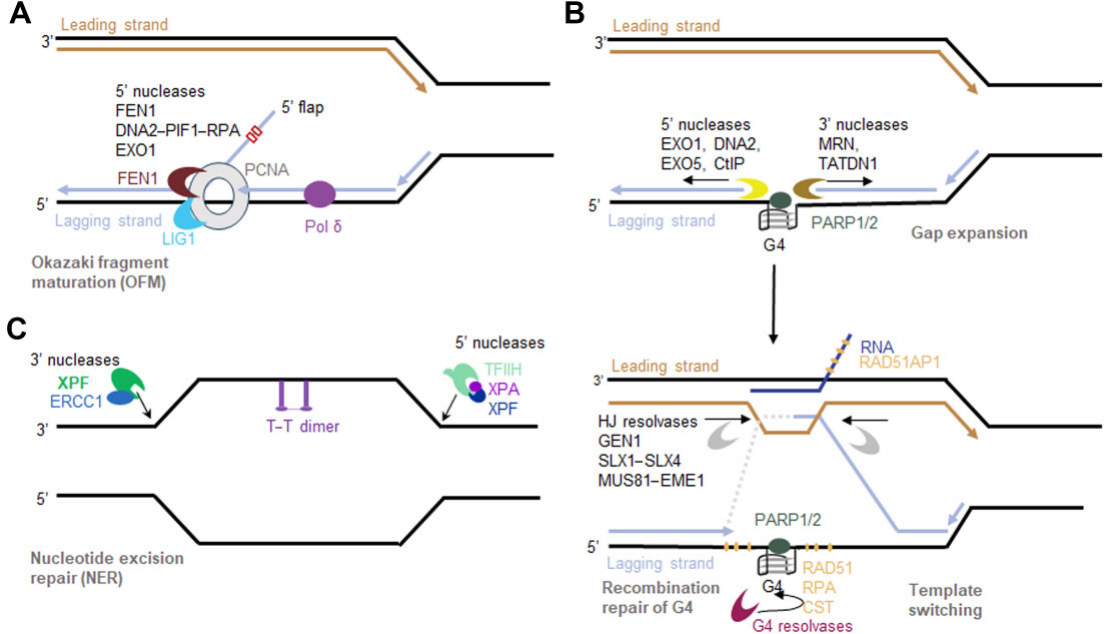
Diagram to show the roles of nuclease complexes in DNA substrate transitions in DNA replication, recombination, and repair pathways. (**A**) During the lagging strand DNA synthesis and OFM, Pol δ displaces the down-strand RNA/DNA primers into a 5′ flap. DNA2/PIF1/RPA complex removes the long flap or flap with secondary structures, while FEN1 removes the short flaps. EXO1 is back up enzyme for OFM. (**B**) Homologous recombination (HR)-mediated repair of gaps during DNA replication. While Pol δ runs into a G4 structure, it jumps over to have a discontinuous synthesis and leaves a gap in the newly synthesized DNA. Several 5′ and 3′ nucleases including EXO1, DNA2, EXO5, CtIP, MRN, and TatD DNase domain containing 1 (TATDN1) are indicated to be involved in expansion of the gap. The 3′ end of the DNA may invade into the part of newly formed sister chromatin to be extended. Then the further end comes back to the original template. At least three other nuclease complexes were indicated to resolve the intermediate Holliday junction to conclude the gap repair. (**C**) The nuclease complexes, XPF/ERCC1 and XPG/TFIIH/XPA in NER to excise the DNA fragment containing the thymidine-thymidine dimers induced by UV. HJ, Holliday junction.

Structure-specific nucleases play essential roles in three key processes at interface of OFM and DSB recombination repair, including removal of the RNA primers as intermediate substrates of lagging strand DNA synthesis, DNA end resections to generate 3′ ssDNA for its sister chromatin invasion as well as Holliday junction resolution. The first process is the RNA primer removal. OFM occurs in the final steps of DNA replication where the 5′ RNA primer used to begin DNA synthesis is cleaved by the 5′ flap endonuclease, FEN1, or by Exonuclease 1 (EXO1) (Figure 2A) to allow ligation of the newly synthesized strand. OFM begins by the displacement of the RNA primer into a 5′ flap through upstream DNA synthesis by the DNA polymerase Pol δ. The 5′ flap is then cleaved by FEN1 so that DNA ligase I may ligate the Okazaki fragments together. However, in an alternative pathway, PIF1’s helicase activity can create a 5′ flap of >25 nucleotides allowing the ssDNA-binding protein, RPA, to stabilize the flap structure and inhibit FEN1 activity. As such, DNA2 will first cleave the 5′ flap into a shorter flap that FEN1 is able to recognize. Moreover, in low concentrations or in the absence of FEN1, EXO1, a 5′ exonuclease may substitute the role of DNA2 or FEN1 during OFM (Liu et al., 2016; Figure 2A). However, instead of creating a single nick to remove the flap, EXO1 chews individual nucleotides starting from the 5′ end until it reaches the double-stranded junction.

Proper OFM is necessary to prevent excessive mutations in the genome. For example, in the defective pathway in *Saccharomyces cerevisiae* where Rad27, an FEN1 homologue, is deleted, there is a strong increase of unprocessed DNA flaps and a slight increase in point mutations from Pol α. Moreover, *Rad27*Δ yeast cells showed permissive, normal growth at 30°C but lethality at 37°C, suggesting conditional lethality (Tishkoff et al., 1997). After extended incubation at 37°C, some cells revert to nearly normal growth (Sun et al., 2021). The restrictive temperature of 37°C activates the cell cycle check point protein Dun1 to transform unprocessed 5′ flaps into 3′ flaps (Figure 2A). 3′ flaps have an active 3′-OH group that can act as a nucleophile and anneal to complementary single-stranded regions on the same strand or of a template strand of DNA or even invade into the sister chromatin. The annealed 3′ end is then extended to fill in the gap for a sealable nick. Alternatively, the 3′ flaps could be removed by 3′ flap endonucleases such as xeroderma pigmentosa complementation group F/excision repair cross-complementation group 1 (XPF/ERCC1) and mutagen-sensitive 81 (Mus81) in absence of 5′ flap endonucleases for the sake of cell survivorship (Tishkoff et al., 1997).

The second process is the nuclease-mediated end resection for HDR. Like the exogenous insults from the environment such as ionized radiation, separation of the DNA double-strand molecule to form replication fork and discontinued synthesis of the lagging strand DNA result in high risk of DSBs. DSBs are seriously harmful genomic lesions that threaten genomic stability and cell survival (Gennery et al., 2000; McKinnon, 2009; White and Vijg, 2016). DNA end resection initially generates 3′ ssDNA, which provides a platform for recruiting HDR-related proteins (Huertas, 2010). For the initiation of DNA end resection, CtBP-interacting protein (CtIP) functions together with the MRE11–RAD50–NBS1 (MRN) nuclease complex to generate a short ssDNA at the DSB ends. After ssDNA is generated by the CtIP/MRN complex, downstream nucleases and helicases, such as EXO1 or DNA2 and Bloom syndrome protein (BLM), are recruited to extend the 3′ ssDNA for HR-mediated repair (Huertas and Jackson, 2009; Yun and Hiom, 2009). In other cases, DNA replication machinery runs into special genome regions such as the telomeres and centromere, where secondary structures known as G-quadruplexes (G4) form when the DNA duplex opens, posing a challenge for lagging strand DNA synthesis and OFM (Li et al., 2018). In this situation, daughter lagging strands are sometimes synthesized in a trans-lesion manner as there is missing genetic information contained in the unresolved secondary structures. To continue DNA replication without loss of genetic information, the undamaged sister chromatid is used as a template through a process called template switching, which is similar to the recombination repair process, requiring an end resection process to generate 3′ flap DNA (Wong et al., 2021), which is coated by ubiquitinated PCNA and Rad51 so that it is protected from nuclease cleavage. The protected 3′ flap is then able to invade the sister chromatin to initiate HDR (Thakar et al., 2020; Figure 2B). Template switching from resolving G4 complexes can result in four-strand DNA intermediates, called Holliday junctions (Figure 2B).

Structure-specific nucleases are also important in Holliday junction resolution. Single or double Holliday junctions formed during HR in DNA repair and inter-homologue pairing in meiosis need to be resolved to prevent non-disjunction, aneuploidy, cell death generation of micronuclei or multinucleate cells (West et al., 2015). This structure forms as an alternative to strand displacement annealing for DNA break repair and represents a means of repair that does not risk alteration of genetic information like in non-homologous end joining. Holliday junction structures are processed through ‘resolution’ or ‘dissolution’. Usually, double Holliday junctions will be dissolved in a 2-step ATP-dependent reaction that uses the BLM helicase–topoisomerase 3a–RM1–RM2 complex. Products of dissolution are distinctively non-crossover, promoting heterozygosity in mitotic cells (Matos and West, 2014). When Holliday junction dissolution fails to occur, a group of ubiquitous and highly specialized structure-selective endonucleases called Holliday junction resolvases catalytically resolve Holliday junctions through the cleavage of the structure into two separate DNA duplexes. These enzymes have been identified widely across all domains of life (Wyatt and West, 2014; Yan et al., 2020). Failure of dissolution or resolution of Holliday junctions between sister chromatids results in aneuploidy, which is common in cancer cells. Moreover, resolution of Holliday junctions can cause loss of heterozygosity due to rare crossover events between homologues.

Precise resolution of stalled or damaged DNA replication forks, replication fork reversal and restart is the core mechanism for high-fidelity DNA replication and genome integrity. The nuclease activity of DNA2 is required to prevent fork reversal. Consistent with this, DNA2 can efficiently cleave the leading or lagging strands of the obligated precursors of fork regression on the replication fork in yeast (Hu et al., 2012). A novel DNA2- and WRN-dependent mechanism of reversed replication fork processing and restart after prolonged genotoxic stress has also been identified in human cells. The human DNA2 nuclease and WRN ATPase activities functionally interact to degrade reversed replication forks with a 5′→3′ polarity and promote replication restart, thus preventing aberrant processing of unresolved replication intermediates (Cejka et al., 2010; Hu et al., 2012; Thangavel et al., 2015).

Dissolution using endonucleolytic resolvases is the primary mechanism to disentangle single Holliday junctions and undissolved double Holliday junctions early in the cell cycle before mitosis. These enzymes recognize flaps and branched structures, including Holliday junctions, as their substrates. Despite displaying poor activity, MUS81/essential meiotic structure-specific endonuclease 1 (EME1/EME2), and potentially structure-specific endonuclease subunits SLX1/SLX4, cleave 3′ flaps and creates two asymmetrical, un-ligatable nicks in the Holliday junction (Schwartz and Heyer, 2011; Piazza et al., 2017; Figure 2B). Differing slightly in activity, flap endonuclease GEN homologue 1 (GEN1) and SLX1/SLX4 cut 5′ flaps while making two symmetrical nicks (Schwartz and Heyer, 2011; Figure 2B). However, in the absence of resolvases MUS81 and GEN1, sister chromatids have unresolved Holliday junctions that result in HR-generated ultrafine anaphase bridges in mitosis, leading to mild impairment of sister chromatid separation (Chan et al., 2018). Ultrafine anaphase bridges are a product of BLM helicase and PICH processing of ssDNA, RPA coating, and rupturing in mitosis. In an alternative pathway, XPF and three-prime repair exonuclease 1 (TREX1) proteins have a role in reducing the link between chromatids to a single strand (Sasaki and Tonomura, 1973). None of these events trigger checkpoint responses until the subsequent cell cycle and a few cells (∼2%) may survive with chromosomal abnormalities. These findings suggest a real need for the action of resolvases during mitosis to promote the dissolution pathway of Holliday junctions. The effect of GEN1 and MUS81 loss would qualify as a synthetic lethal condition as loss of either barely perturbs cancer-derived cell line growth or survival.

TREX1 is the most abundant 3′→5′ DNA exonuclease and RNA exonuclease in mammalian cells (Perrino et al., 1994; Yuan et al., 2015). In various autoimmune diseases, dysfunctional TREX1 leads to accumulation of endogenous ssDNA, dsDNA, and DNA/RNA hybrids in the cytoplasm and triggers immune activation. Mutations in TREX1 cause autoimmune disorders, including Aicardi–Goutieres syndrome type 1, systemic lupus erythematosus, chilblain lupus, and retinal vasculopathy with cerebral leukodystrophy (Yuan et al., 2015; Wang et al., 2022). Suppression of TREX1 activity has been indicated as a strategy for cancer immunotherapy (Hemphill et al., 2021). Nevertheless, its cellular roles in various biological pathways are pending for current and future studies, particularly, on its functionality in processing DNA/RNA hybrids and RNA substrates (Huang et al., 2023). The other three-prime repair exonuclease, TREX2, is involved in maintenance of the genome stability and suppression of mutations generated in replication fork in HR-defective cells (Hasty, 2021). Both TREX1 and TREX2 play an important role in cellular apoptosis DNA fragmentation (Chowdhury et al., 2006; Cheng et al., 2018; Zhou et al., 2022; Chen et al., 2023).

## Nucleases in DNA excision repair in response to exogenous DNA damage

The genome is consistently maintained by structure-specific nucleases through multiple DNA repair pathways, particularly in the non-S phase cells. Structure-specific nucleases are essential components for all DNA repair pathways. One of the most efficient mechanisms for repairing helix-distorting DNA lesions caused by UV irradiation or platinum-based antitumour agents is nucleotide excision repair (NER). Two structure-specific endonucleases, XPG (*ERCC5*) and XPF (*ERCC4*), are crucial in removing all DNA fragments that contain the lesions (Figure 2C). Preclinical model studies reveal that most cisplatin crosslinks formed on DNA are recognized and repaired by the mammalian NER apparatus (Rabik and Dolan, 2007; Graf et al., 2011). NER is a multistep process, involving some 20 different genes. Among these genes, *ERCC1* and the seven Xeroderma Pigmentosum (XP) genes, *XPA* to *XPG*, play critical roles in damage recognition, demarcation, and strand incision around the lesion site. In the NER pathway, a 24–32 nucleotide incised strand containing the DNA lesion is removed and the resulting gap is subsequently filled with dNTPs by DNA polymerase using the complementary strand as a template and sealed by DNA ligase (Graf et al., 2011). The endonuclease XPG cuts 5–6 nucleotides downstream (3′) of the DNA damage, while ERCC1–XPF cuts 20–22 nucleotides upstream (5′) of the DNA damage (Friedberg, 2001; Leibeling, et al., 2006; Graf et al., 2011). The presence of either XPG or the 3′ incision made by XPG is a prerequisite for the 5′ incision activity of XPF (Pal et al., 2022).

Small types of damage such as deamination, oxidation, or methylation to nitrogenous bases are repaired through the base excision repair (BER) pathway. Long patch BER is very similar way to NER. The primary nuclease, apurinic/apyrimidinic endonuclease 1 (APE1), makes a nick in the 5′ side of the damaged base site, generating an ssDNA fragment as a flap, which is then removed by FEN1 (Dianov and Lindahl, 1994; Nicholl et al., 1997; Klungland and Lindahl, 1997; Srivastava et al., 1998; Pascucci et al., 1999). Similarly, the damaged nucleotide in the newly synthesized DNA is excised in the 3′ manner by the 3′ nuclease activity of DNA polymerases, such as Pol δ and Pol ε, or in the 5′ manner by FEN1 and EXO1. In all these excision pathways, eukaryotic cells have evolved to avoid formation of the active 3′ flap presumably because it can cause genome rearrangements and instability through strand invasion.

DNA inter-strand crosslinks (ICLs) are regions of the DNA that cannot be separated into single strands due to covalent bonds, thereby preventing replication and transcription. The Fanconi anemia (FA) repair pathway promotes the excision of ICLs using at least 22 FANC proteins, including FANCS/BRCA1 and FANCD1/BRCA2 (Kottemann and Smogorzewska, 2013; Wang and Smogorzewska, 2015; Ceccaldi et al., 2016). Absence of the FA pathway may lead to developmental abnormalities, bone marrow failure and cancer predisposition, primarily acute myeloid leukaemia, and squamous cell carcinomas (SCC) of the aerodigestive tract (Alter et al., 2018; Webster et al., 2022). The FA pathway is thought to coordinate a complex mechanism that enlists elements of three classic DNA repair pathways, HR, NER, and mutagenic translesion synthesis, in replication-dependent repair of ICLs, the latter has been established (Chowdhury et al., 2006; Cheng et al., 2018). A unique nuclear protein complex in the FA pathway ubiquitinates FANCD2 and FANCI, leading to formation of DNA repair structures (Zhao et al., 2024). Recently, it has been demonstrated that the primary genomic signature of FA repair deficiency is the presence of high numbers of structural variants including small deletions, unbalanced translocations, and fold-back inversions, forming complex rearrangements (Webster et al., 2022).

During DNA replication in the S phase cells, replication forks converging from different directions stall at the ICL site ∼20–40 nucleotides before the lesion. Subsequently, one fork moves forward and stops just before the lesion. Dual incision on each side of the ICL, likely performed by two structure-specific nuclease complexes, Mus81–Eme1 (the proximal) and Ercc–XPF (the distal), excise the ICL, which is then bypassed by a TLS polymerase, likely Rev1. Pol ζ then extends from the initial insertion, and the NER system removes the bypassed crosslink. The HR machinery including Fanconi-associated nuclease 1 (FAN1) then repairs the broken chromatid using the newly repaired sister as a template to complete the process (Moldovan and D'Andrea, 2009; Raya et al., 2009).

FA itself is a genome instability disorder that results in excessive chromosome breakage due to a deficiency in DNA ICL repair (Sasaki and Tonomura, 1973; Auerbach and Wolman, 1976; Yang, 2011; Zheng and Shen, 2011; Zhou et al., 2015; Taylor et al., 2019). More specifically, the FA repair pathway protects against endogenous and exogenous carcinogenic aldehydes that might result in ICLs (Reardon and Sancar, 2003; Nick McElhinny et al., 2008; Pontel et al., 2015; Garaycoechea et al., 2018; Rycenga and Long, 2018; Zheng et al., 2020; Bousios et al., 2020). Individuals with FA are more likely to develop head and neck (HNSCC), esophageal, and anogenital SCC on the magnitude of hundreds to thousands (Alter et al., 2018). While it is known that HNSCCs are primarily driven by tobacco and alcohol exposure with and without infection with HPV, it is still unclear how HNSCCs are related to SCCs from individuals with FA (FA SCCs) due to limited molecular studies (Nagarajan et al., 2013; Johnson et al., 2020). The genomic instability in sporadic HPV-negative HNSCC may arise as a result of the FA repair pathway being overwhelmed by DNA ICL damage caused by alcohol and tobacco-derived aldehydes, making FA SCC a powerful model to study tumorigenesis resulting from DNA ICL damage (Webster et al., 2022).

The remaining characterized DNA excision repair pathway is DNA mismatch repair (MMR), in which the action of nucleases has been known for a long time. FEN1 or its yeast homologue RAD27 was initially identified as an MMR nuclease because of its strong mutator phenotype (Johnson et al., 1995). It was later on realized that the FEN1’s strong mutator phenotype is due to its role in OFM (Tishkoff et al., 1997). Then, the field has turned the attention to EXO1 despite its weak mutator phenotype, compared with other MMR components such as MSH2 (Tishkoff et al., 1998; Calil et al., 2021; Kadyrova et al., 2022). Exo1 meets all criteria in such an important DNA replication-coupled excision repair pathway as its deficiency diversifies the neoantigen and benefits the immunotherapy cancer patients (Cortez, 2019; Vali-Pour et al., 2022; Wang et al., 2022).

## Nucleases in apoptotic DNA fragmentation

**Figure 3:**
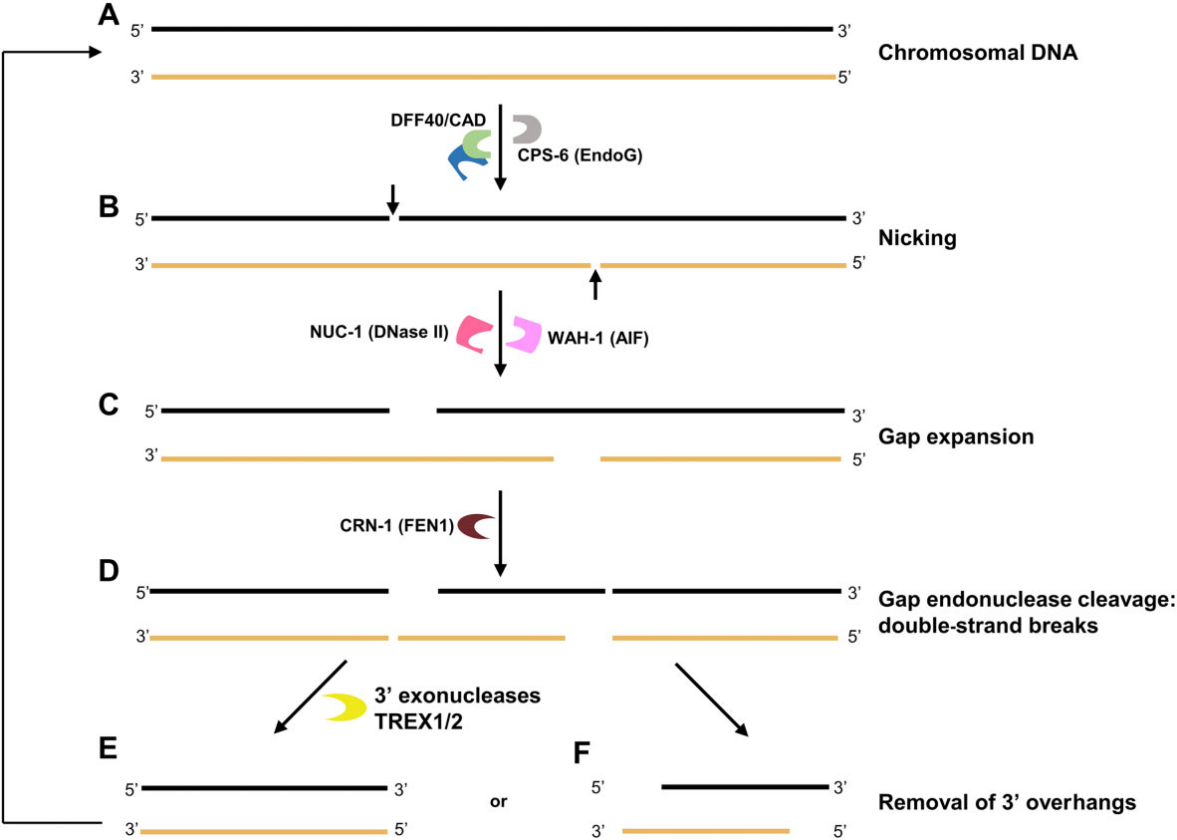
Molecular model for nucleases-mediated chromosome fragmentation during apoptosis. (**A**) Chromosomal DNA. (**B**) DNA is nicked by DDF40/CAD and/or *C. elegans* homologue CPS-6 of human EndoG. (**C**) Following nicking, the 5′→3′ exonuclease activities of NUC-1 (DNase II) and WAH-1 (AIF) turn the nicks into gaps. (**D**) The resulting gapped substrates are cleaved by FEN1/CRN-1 gap-dependent endonuclease activity (aided by CPS-6), resulting in DSB to produce the fragmented substrates. (**E**) These fragmented substrates can be either further processed by a 3′→5′ exonuclease(s) such as TREX1/2 to remove 3′ overhangs (**F**) or directly processed through similar steps (**A**–**D**) to generate smaller DNA fragments.

Fragmentation of chromosomal DNA is a necessary stepwise process in the apoptotic pathway to prevent autoimmune reactions to excess circulating DNA. In mammalian cells, *DFFB* gene-encoded 40-kDa DNA fragmentation factor/caspase-activated deoxyribonuclease (DFF40/CAD) associates with nuclear proteins to initiate inter-nucleosomal DNA cleavage, while mitochondrial endonuclease G (EndoG) translocates from mitochondria to the nuclei to promote a caspase-dependent and DFF40-independent fragmentation pathway (Liu et al., 1997; Enari et al., 1998; Widlak et al., 2001). The activity of the human nuclease, EndoG, on DNA can be stimulated by the nucleases, deoxyribonuclease I (DNase I) (bovine), and ExoIII (*Escherichia coli*) *in vitro* (Widlak et al., 2001). Similarly, in *Caenorhabditis elegans*, disturbing either the exo- or endo-nuclease activity of CRN-1 (cell death related nuclease-1, an FEN1 homologue) will reduce CPS-6 (EndoG homologue) expression (Harrington and Lieber, 1994; Bambara et al., 1997; Lieber, 1997; Figure 3). Like FEN1, CRN-1 plays a role in DNA repair and replication. Upon association with CPS-6, CRN-1 transforms into a genome destroyer in preparation for DNA fragmentation. Moreover, CPS-6 can increase 5′→3′ exonuclease activity and gap-specific endonuclease activity of CRN-1 but fails to increase the flap endonuclease activity limited to DNA replication and repair functions (Parrish et al., 2003). Therefore, CPS-6 and CRN-1 together can initiate and facilitate DNA fragmentation during apoptosis (Figure 3). First, CPS-6 and other similar nucleases will start by generating nicks in dsDNA (Parrish et al., 2003). The ssDNA nicks will expand into ssDNA gaps via NUC-1 (DNase II) and WAH-1 (AIF) 5′→3′ exonuclease activity, followed by dsDNA breaks caused by CRN-1 gap-specific endonuclease activity. Figure 3 illustrates how several key nucleases among others work cooperatively to promote a stepwise apoptotic DNA degradation pathway (Parrish et al., 2003).

Identification of several other candidate genes involved in DNA fragmentation for apoptosis in *C. elegans* was performed using RNA interference (RNAi). Of 77 open reading frame candidates (nucleases and non-nucleases), 9 were subsequently identified to have genes encoding nuclease activities in apoptotic pathways. Genes of the indicated nucleases were silenced with RNAi treatment of larvae at various stages. The surviving progeny were screened for DSBs by labelling the 3′-hydroxyl termini via terminal deoxynucleotidyl transferase dUTP nick end labelling (TUNEL). Most viable RNAi-oligo-treated progeny had TUNEL-positive nuclei. Each of the genes yielding TUNEL positive phenotypes when silenced were then selected for further testing to determine possible cooperation among candidates in promotion of apoptosis. For instance, when *CRN-1* RNAi was treated to *CPS-6* mutant worms, the TUNEL phenotype did not seem to intensify, suggesting that *CPS-6* and *CRN-*1 are involved in similar roles during apoptosis, consistent with the proposal of CPS-6 recruiting CRN-1 for apoptosis. The positive interactions among candidates (*cps-6, nuc-1, crn-1, crn-2, crn-3, crn-4, crn-5, crn-6*, and *cyp-13*) and identification of two pathways verified the involvement of the additional nucleases (Parrish and Xue, 2003).

## Nucleases in the HIV life cycle

**Figure 4:**
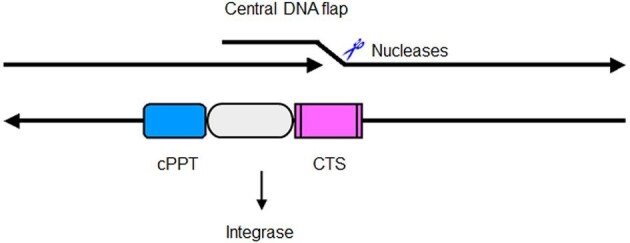
The plus strand of the HIV viral genome is synthesized by its reverse transcriptase using virion RNA as a template. The minus DNA strand is synthesized discontinuously: the first strand (upstream) is synthesized and terminated at the CTS, while the second strand (downstream) starts at the cPPT to be extended to the end, which results in a three-strand region with a CDF. A flap endonuclease either 3′ or 5′ nuclease like FEN1 is required to remove such a structure before the viral genome may be integrated into the host genome.

Structure-specific nucleases play an important role in viral genome dynamics and integration into the host genomes. HIV-1 and other lentiviruses have a complex reverse transcription strategy, that is, there are two additional *cis*-acting sequences within the lentiviral genome, namely, the central polypurine tract (cPPT) and the central termination sequence (CTS), which leads to the formation of a triple-stranded DNA structure, the central DNA flap (CDF) (Hameau et al., 2001; Dvorin et al., 2002; De Rijck et al., 2005; Arhel et al., 2006; Figure 4). The CDF acts as a *cis*-determinant of HIV-1 DNA nuclear import (Zennou et al., 2000; Ao et al., 2004). Wild-type viral linear DNA is almost entirely imported into the nucleus where it integrates or circularizes. In contrast, mutations within cPPT lead to linear genome DNA accumulating in infected cells, at the vicinity of the nuclear membrane, indicating a late defect in nuclear import, which profoundly impairs HIV replication and infectivity (Arhel et al., 2006). Accordingly, reinsertion of the DNA flap in HIV-1 vectors significantly stimulated gene transfer efficiencies both *in vivo* and *ex vivo* in all tissue- and cell-types examined (Poeschla, 2013).

The CDF is 99 nucleotides in length and located in the gene encoding HIV-1 integrase. It separates the cPPT and the CTS (Poeschla, 2013). The process of CDF formation is mediated by a large nucleoprotein complex termed the pre-integration complex (PIC) (Bukrinsky et al., 1992; Miller et al., 1995; Lusic and Siliciano, 2017). Viral DNA with this overlap structure facilitates integration. In the bona fide viral context, it is apparently not found following transport to the nucleus, suggesting the CDF structure, being an intermediate product of the HIV lifecycle, must be rapidly repaired by normal cellular mechanisms to generate a stable viral genome for PIC nuclear import and viral DNA integration (Miller et al., 1995).

Processing the CDF is, in part, a DNA repair process and may involve nuclease(s). Thus far, no viral protein or host factor has been identified to cleave such a DNA structure *in vivo* (Poeschla, 2013). Human cellular FEN1 is a likely candidate to process the overlap region because of its substrate specificity (Rumbaugh et al., 1998). FEN1 is a nucleic acid substrate structure-specific nuclease that possesses 5′ flap endonuclease activity, allowing cleavage of the 5′ flap DNA structure formed during lagging strand DNA synthesis and base excision repair (Liu et al., 2004; Shen et al., 2005). It also possesses a novel gap-specific endonuclease activity more important for this pathway (Parrish et al., 2003; Zheng et al., 2005). This activity enables the enzyme to cleave the 5′ flap strand with stable secondary structures (Parrish et al., 2003; Zheng et al., 2005). The biochemical property by which FEN1 actively resolves triple-stranded DNA structures suggests that the enzyme may be involved in processing the HIV-1 CDF. Indeed, a previous study has shown that recombinant hFEN1 cleaves a synthetic HIV-1 flap model substrate resembling the exact HIV nucleotide sequence *in vitro* (Rumbaugh et al., 1998). It is unknown whether FEN1 plays a role in repairing HIV-1 CDF *in vivo*. Furthermore, despite studies examining the effects of mutated/deleted CDFs, or cases where the formation of CDFs was abolished on nuclear import of both viral DNA (Iglesias et al., 2011) and viral replication (Ao et al., 2004; De Rijck and Debyser, 2006; Marsden and Zack, 2007), the biological consequence of not cleaving the HIV-1 CDF remains unclear. In our published patent (Shen and Zheng, 2006), we revealed that blockage of HIV-1 CDF cleavage can inhibit the proper transcription/translation of HIV-1 integrase and thereby suppress viral replication. Thus, HIV-1 CDF processing may represent a novel target for anti-HIV therapeutic intervention.

## Would the nucleases be good anti-cancer targets?

A major disadvantage of conventional chemotherapy is its lack of cancer cell specificity. Identifying pathways that specifically support cancer cells is key for effective treatment. Cancer cells with activated oncogenes, aberrant checkpoints, and/or aneuploid/polyploid genomes have aberrant DNA replication, which manifests as a high level of DSBs due to stalling and collapse of DNA replication forks (Bartkova et al., 2006; Negrini et al., 2010; Zheng et al., 2012; Gaillard et al., 2015; Macheret and Halazonetis, 2015). Hormone-related cancers such as breast, ovarian, and prostate cancers have an additional DSB burden, because estrogen/androgens induce hyper-activated signalling to provoke excessive DNA replication and cell proliferation, as well as trigger robust transcription, resulting in both replication- and transcription-associated DSBs (Haffner et al., 2011). Given that a single unrepaired DSB may cause cell death (Bennett et al., 1993, 1996), most chemotherapies also target replication forks to directly or indirectly induce DNA DSBs (Cannan and Pederson, 2016). Targeting both DNA replication and DNA repair has recently been proposed as a major opportunity to enhance current cancer therapies based on DNA damaging agents by blocking DSB repair (Helleday et al., 2008; Somaiah et al., 2012). DNA repair-targeted therapy is rapidly growing; to date, promising targets include ∼20 DNA repair enzymes and other key proteins that inhibit DNA DSB repair or other repair pathways (Gavande et al., 2016).

**Figure 5:**
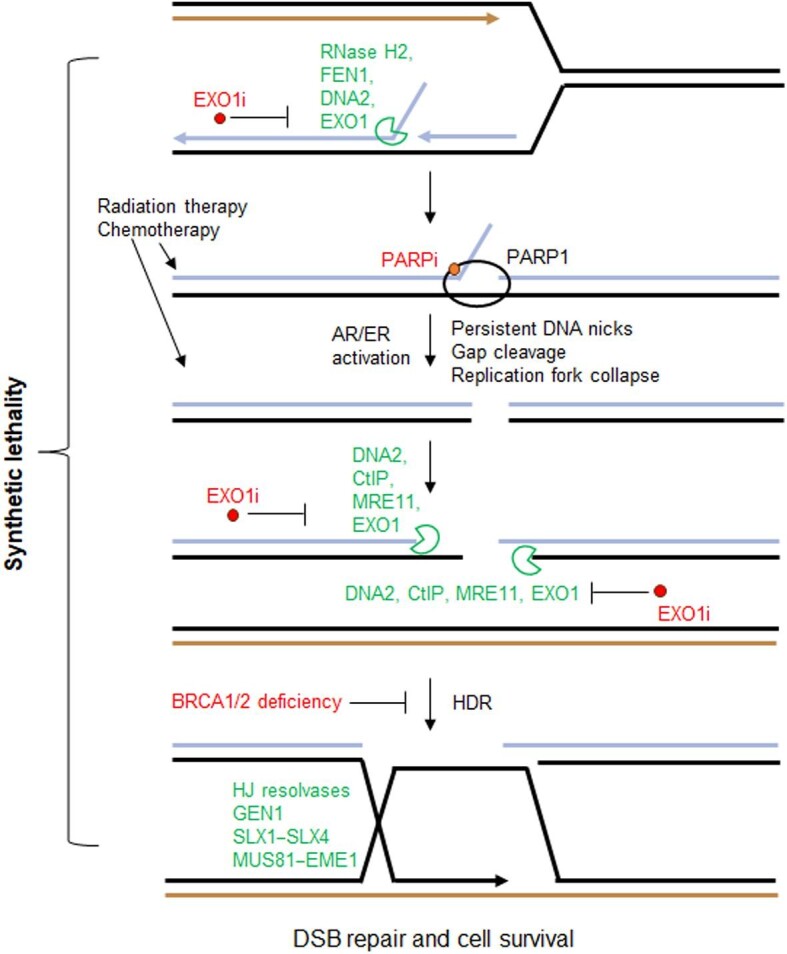
Working model elucidating dual functions of EXO1 inhibition for blocking cancer cell survival. Inhibition of EXO1 impairs OFM, leading to accumulation of single-strand breaks and subsequently DSBs. Meanwhile, EXO1 inhibition inhibits DNA end resection for DSB repair. Altogether, EXO1 inhibition presents a new way to block cancer cell survival via targeting the DNA replication and repair interface, which mimics the synthetic lethality combination of PARPis and a BRCAness genetic background.

Advancements in describing the mechanisms of DNA replication in cancer cells under stress conditions suggest that aberrant DNA replication and repair processes in cancer cells are interconnected. Thus, we propose that one way to maximally harness DSBs in cancer cells for cancer therapy is to target the replication-repair interface that processes replication intermediates and repairs replication-associated DSBs. This will allow simultaneous enhancement of DSB induction and blockage of its repair, thus building up DSBs and exceeding a cancer cell's capacity to counteract or tolerate them. The multifunctional DNA repair enzymes such as DNA2 and EXO1 are key controlling factors in the replication-repair interface (Figure 5). They are two of four important nucleases, i.e. ribonuclease H2 (RNase H2), FEN1, DNA2, and EXO1, involved in OFM, the process by which RNA–DNA flaps are removed to generate ligatable nicks for ligation of individual Okazaki fragments into the intact lagging DNA strand during replication (Zheng et al., 2011, 2020; Zheng and Shen, 2011). If the OFM machinery is inhibited, the persistence of flap/nick structures in the wake of the advancing replication fork represents the most vulnerable phase in the cell cycle, at which time significant DNA replication stresses and DSBs are generated. Inhibition of any of the nucleases abolishes OFM, generating persistent flap/nick substrates in S phase, and subsequently causes DSBs (Zheng et al., 2011, 2020; Zheng and Shen, 2011; Zimmermann et al., 2018). Meanwhile, DNA2 and EXO1 play a crucial role in repairing DSBs, including those derived from collapsed or cleaved replication forks (Figure 5). Four nucleases including EXO1 (Nimonkar et al., 2011), DNA2 (Peng et al., 2012), CtIP (Sartori et al., 2007), and Mre11 (Nimonkar et al., 2011) are largely players responsible for DSB end resection to generate 3′ ssDNA overhangs, which in turn activate the master signalling kinase ATR (Bao et al., 2001; Cortez et al., 2001; Zou and Elledge, 2003) and invade sister chromatin DNA to initiate DSB response and repair (DSBRR) via HDR (Ira et al., 2004; Kakarougkas and Jeggo, 2014). Blocking DNA end resection prevents DSB repair by HDR. Given that cancer cells require EXO1 for counteracting stress due to abnormal DNA replication, small molecules targeting EXO1 represent a major opportunity for specifically killing cancer cells by targeting the aberrant replication-associated repair system in human breast cancer and other hormone-related cancers.

Genetics studies have revealed that cancer cells frequently bear genetic alterations that disrupt the function of DSBRR proteins. Exploiting such genetic alterations in tumours via inhibition of DNA repair pathways including DSBRR is an increasingly attractive strategy for inducing synthetic lethality, in which combined disruption of two genes or pathways leads to cell death (Nijman, 2011). Inducing synthetic lethality by inhibiting replication and repair genes creates cancer cell specificity, because normal cells are well protected from synthetic lethality by normal checkpoints and robust backup DNA repair pathways. Poly (ADP-ribose) polymerase (PARP) inhibitors (PARPis) are one successful example of exploiting synthetic lethality in cancer (Falchi et al., 2017; Lord, and Ashworth, 2017; O'Neil et al., 2017). Convincing clinical data indicate that PARPis improve progression-free survival in hormone-related cancers (e.g. breast, ovarian, and prostate cancer) that are deficient in DSBRR genes such as BRCA1/2, PALB2, BRIP1, and ATM/ATR (Bhalla and Saif, 2014; Lord and Ashworth, 2017; Nientiedt et al., 2017). PARPi-mediated synthetic lethality in DSBRR-deficient cancers is due to inhibitory effects on the catalytic activity of PARP and induction of PARP to ‘trap’ the nick sites of genomic DNA, leading to DSBs in S-phase cells (McCabe et al., 2006; Shen et al., 2013; Steffen et al., 2013; Murai et al., 2014; Konstantinopoulos et al., 2015; Brown et al., 2017; Maya-Mendoza et al., 2018). Efficient repair of PARPi-induced DSBs in S-phase human cells depends on HDR pathways, which require the DNA damage response proteins BRCA1/2 (Zhang et al., 1998; Venkitaraman, 1999), ATM/ATR (Paulovich and Hartwell, 1995), Chk1/2 (Paulovich and Hartwell, 1995; Sanchez et al., 1996; Matsuoka et al., 1998), PALB2 (Xia et al., 2006; Rahman et al., 2007), and BRIP1 (Bridge et al., 2005); the Fanconi Anemia (FA) proteins; and the HDR proteins that include EXO1 (Mimitou and Symington, 2008; Zhu et al., 2008), DNA2 (Zhu et al., 2008; Cejka et al., 2010), and RAD51 (Davies et al., 2001). Gene deficiency in any of these DSBRR genes, or ‘BRCAness’, a phenotype first defined in BRCA1/2-mutated cancers, impairs DSB repair and promotes PARPi synthetic lethality (Turner et al., 2004; Tutt, 2018).

Due to their dual roles in DNA replication and DSB repair, DNA2 and EXO1 are not only two exceptional targets for cancer cell-specific killing, but also two outstanding targets for inducing synthetic lethality of cancer cells. DNA2 and EXO1 inhibitors (DNA2is and EXO1is) would impair OFM and cause persistent ssDNA breaks, with the potential for conversion into DSBs as well as for PARP trapping (Liu et al., 2016; Paiano et al., 2021; Figure 5). This would induce synthetic lethality in human cancers with HDR gene deficiency, e.g. BRCA1/2 mutations, as well as sensitize cancer cells to PARPis. In addition, EXO1i(s) would display greater specificity than PARPis for synthetic lethality with HDR gene deficiency, because PARPs participate in a wide array of other cellular processes such as transcription and translation, whereas EXO1 does not. EXO1 is also itself a crucial enzyme for the HDR process; its deficiency impairs HDR, yielding BRCAness and potential synthetic lethality with PARPi. Thus, EXO1i-induced BRCAness may extend PARPi therapy to larger patient populations without HDR gene mutations. Therefore, selective, and effective DNA2is and EXO1i(s) as examples of structure-specific nuclease inhibitors can be used as both a research tool (i.e. chemical probe) and a pre-clinical starting point towards development of a potential therapeutic drug that induces cancer cell-specific killing via synthetic lethality.

## Strategies to develop nuclease inhibitors for cancer therapy

**Figure 6:**
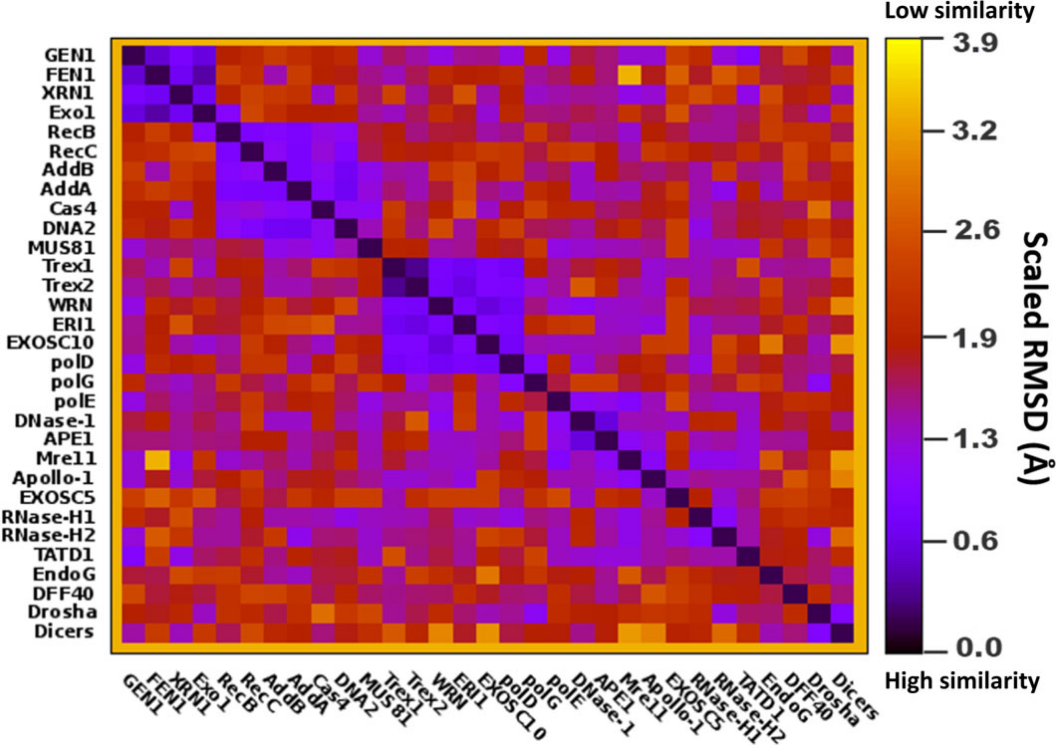
The structural similarity heatmap of nuclease catalytic domains measured by Scaled RMSD in angstrom. 3-dimensional structures of nucleases, including 21 from Table 1, are aligned using a scaled root-mean-square deviation parameter (Scaled RMSD; regular RMSD value divided by the cube root of the aligned residue pair number) to have a simple metric for relative structural similarities. Scaled RMSD, which we developed here to minimize the protein length derivation, more clearly displayed structure similarities within certain clusters of enzymes than template modelling score. This figure was illustrated by Microsoft Excel.

Taking advantage of available knowledge of their diversified structures and functions, the active efforts are being invested in identifying and developing small molecular inhibitors for the therapeutic regimens. Here, we reviewed and aligned 3-dimensional structures of nucleases, including 21 from Table 1, using a scaled root-mean-square deviation parameter (scaled RMSD; regular RMSD value divided by the cube root of the aligned residue pair number), to have a simple metric for relative structural similarities. Scaled RMSD, which we developed to minimize the protein length derivation, more clearly displayed structural similarities within certain clusters of enzymes than template modelling score. As depicted in Figure 6, the overall 3-dimensional structural similarities among the nucleases are low. However, there are four clusters of nucleases with statistically significant similarities. The first cluster with relatively high similarity includes FEN1, GEN1, EXO1, five-prime exoribonuclease 1 (XRN1), and probably XPG, which was not analysed but generally grouped with FEN1, GEN1, and EXO1 as a structurally similar group (Jinek et al., 2011). The second cluster is composed of RecB, RecC, AddB, AddA, Cas4, and DNA2. The third cluster has members including TREX1, TREX2, WRN, exoribonuclease 1 (ERI1), exosome component 10 (EXOSC10), and the nuclease domain of Pol δ. The last cluster, with lower similarity to group 1, includes the nuclease domain of Pol ε, DNase I, APE1 (*APEX1*), MRE11, and Apollo-1 (*DCLRE1B*). Based on the uniqueness of the structures and functions of the various structure-specific nucleases, we expect that the inhibitors development against them would be specific with little off target effects.

For catalytic centres of FEN1 and two other metallo- nucleases, the scaled RMSD metric for FEN1 (PDB ID 3q8k) and EXO1 (PDB ID 5v06) is 2.9 Å, while the structures of FEN1 and DNA2 (homology model from PDB ID 5ean) have quite different nuclease domains; RMSD = 6.4 Å. All three nucleases have a pair of magnesium ions that clip the DNA molecule. The metal atoms are coordinated by three to five acidic, negatively charged glutamate and aspartate residues. As shown in Figure 1, the two magnesium ions in the catalytic centre of FEN1 are coordinated by D86, E158, E160, D179, and D181. In EXO1, the pair of metal atoms are coordinated by D78, D152, D171, and D173, while in DNA2, the catalytic residues are H163, D277, and E298. A general examination of documented metal-binding compound inhibitors of a wide range of metalloenzymes, showed that even at a concentration of 10 μM, off-target effects occurred usually where the metalloenzymes shared the same catalytic metal, structure, and general function (Day and Cohen, 2013). Thus, off-target effects are unlikely between FEN1 and DNA2, while possible within the FEN1/GEN1/XPG/XRN1 cluster. Even here, discrimination was observed for FEN1 and XPG, particularly after modification of lead FEN1 inhibitors based on *n-*hydroxyurea compounds (Tumey et al., 2005). Metal chelation inhibitors do not seem to pose greater risks for non-specific metal chelation than off-target effects of other classes of inhibitors, despite various combinations of the metal binding groups (MBG) and backbone. Metal-chelating metalloprotein inhibitors continue to be contemplated as therapeutic drugs in treating cancers and other diseases.

**Figure 7:**
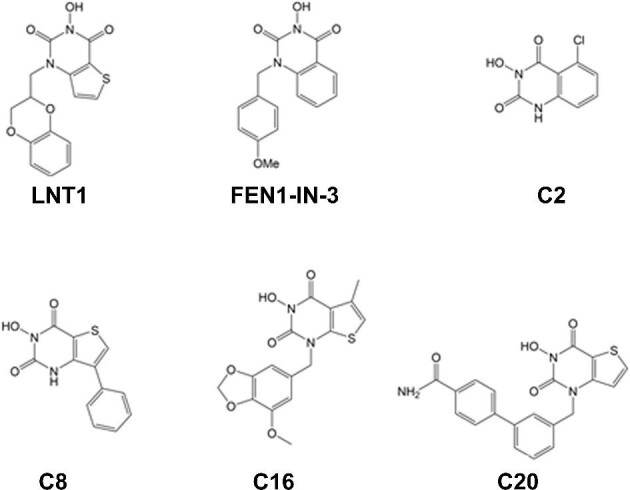
A strategy to develop Mg^++^ contained nuclease inhibitors. The inhibitors with *n-*hydroxyurea utilize a hydroxyl of the inhibitor compounds to replace amino acid interactions by clustering together with magnesium cofactors. All the molecular formulas of this figure were drawn by ACD/ChemSketch.

The range of MBGs includes hydroxamic acid, carboxylate, hydroxypyridinonate, thiol, phosphonate, and *n*-hydroxyurea functional groups, in order of usage in studied inhibitors (Protein Data Bank). For example, the inhibitors with *n-*hydroxyurea utilize a hydroxyl of the inhibitor compound to replace amino acid interactions by clustering together with magnesium cofactors (Figure 7). These MBG are attached to a backbone group with a linker. The backbone groups consist of assorted substituted aliphatic chains, aromatic rings, and heterocycles selected based on compatibility with the conformation of the active site. In contrast with the wider range of available backbone groups, MBG groups are much more limited. This is problematic, as hydroxamic acids (Coussens et al., 2002; Fisher and Mobashery, 2006) and thiols have limited pharmacokinetic properties, leaving only a few MBGs available for the development of new drugs (Lin and Lu, 2001). Another avenue of metallo-inhibitor development found that flutimide-like structures inhibited FEN1 (Tumey et al., 2005). Ideaya Biosciences then developed a similar heterocyclic ring system that restructured a carbonyl of the *n*-hydroxyurea residue to rearrange its position of approach in an effort to optimize chelation (Coussens et al., 2002).

## Conclusion, remarks, and prospective

After 30 years of work, the structure-specific nature of the eukaryotic nucleases is still fascinating to us. That nature has a profound sense to evolve the cells to create the nucleases that recognize the DNA/RNA substrate configurations instead of the sequences as the sequence motifs are fixed in placed and damage occurs in the genomes regardless of the sequence contents. The knowledge made available based on the studies of structure-specific nucleases and their roles in DNA replication and repair is useful to explain important puzzling observations. We were able to predict that FEN1 is the enzyme which is responsible for the HIV CDF processing and suppression of the FEN1 activity may stop the HIV life cycle. In the other example, for years, researchers tried to identify the ‘hot spots’ or specific sequences of the HIV viral integration to the human genome but failed. Even though the HIV integrase shares little amino acid sequence similarity to any of the structure-specific nucleases described here, its 3-D structural topology and predicted molecular actions mimic FEN1 (Finger et al., 2012). We also speculate that the HIV integrase binds and scans the human genome to search for the abnormal DNA structures as sites of viral genome integrations. It looks for the abnormal DNA structures regardless of the sequence motifs or genomic locations. For a long time, it was observed that HBV infection and intake of aflatoxin are two major epidemiological factors for liver cancer (Cunha et al., 2019; Magnussen and Parsi, 2013). They may be linked as we hypothesize that aflatoxin serves as a genome flag for viral integration.

As we place the nucleases as corner stones for the genome dynamics pathways, it becomes clear that deficiency of the nucleases involved in DNA replication and its coupled repair such as MMR display spontaneous strong mutator phenotypes, while nuclease deficiency in the damage induced DNA repair pathway may show weak mutator phenotype unless there are overlap enzymes. Therefore, we speculate there are additional nucleases that serve as excision scalpels for MMR. In vitro biochemical assays of the nuclease activities and configuration specificity are powerful to place the nucleases in a specific pathway (Zheng et al., 2011). However, the combined approaches with cell and structural biological methods as well as genetic analysis will speed up our realization of the goals.

Since the identification of synthetic lethality between BRCA1/2 deficiency and PARP inhibition in 2005, work on finding similar combinations involving nucleases has gained traction. More work is required to establish the networking of nucleases within the DNA replication and repair pathways and in RNA metabolism to achieve successful therapies and clarify possible compensating factors that might thwart or contraindicate use of nuclease inhibition as therapy for a particular cancer (Abbotts et al., 2014; Tomecki et al., 2014; Ward et al., 2017; Chan et al., 2018; Guo et al., 2020; van Wietmarschen et al., 2020; Alvarez-Quilon et al., 2020; Zatreanu et al., 2021; Alblihy et al., 2022). Pathway analysis is sometimes quite involved as multiple nucleases work in the same step of DNA replication, repair, recombination, or apoptosis pathways, yet are not completely redundant. One explanation is that they may work on different regions of genomes and, better documented, in connection with fully functioning replication forks vs. paused or stalled replication forks. For example, during whole mammalian genome replication processes, DNA2 is the nuclease responsible for cleavage of RNA primers with possible secondary structures in difficult-to-replicate regions, such as centromeres and telomeres, while FEN1 is generally responsible for flap cleavage in regions with less stalling or slowing of the replication forks in association with modification-free PCNA-linked processivity (Li et al., 2018). Therefore, FEN1 or DNA2 inhibitors can induce synthetic lethality with BRCA1/2 deficiency and/or with PARPi in human cancer cells (Liu et al., 2016; Mengwasser et al., 2019; Guo et al., 2020).
